# Breath test using ^13^C methacetin does not seem to be useful in the assessment of liver function in girls with anorexia nervosa: a case control study

**DOI:** 10.1186/s12876-018-0856-1

**Published:** 2018-08-13

**Authors:** Katarzyna Górowska-Kowolik, Agata Chobot, Jarosław Kwiecień

**Affiliations:** 1 Department of Pediatric Gastroenterology and Hepatology, Clinical Hospital No 1 in Zabrze of the Medical University of Silesia in Katowice, Katowice, Poland; 2 Department of Pediatrics, School of Medicine with the Division of Dentistry in Zabrze, Medical University of Silesia, Katowice, Poland

**Keywords:** Methacetin breath test, Anorexia nervosa, Liver function, Cytochrome P450

## Abstract

**Background:**

Anorexia nervosa (AN) concerns approximately up to 1.8% of the pediatric female population. One of the complications that can occur in the course of this disease is acute liver failure. This study’s objective was to assess the usefulness of the ^13^C labeled Methacetin Breath Test (MBT) in the diagnostics of the liver function in girls with eating disorders.

**Methods:**

For the study 81 girls aged 12 to 17 years were recruited, including 41 patients with confirmed diagnosis of AN (mean age 14.7 ± 1.48 years) and 40 age-matched controls. The diagnosis was based on the present Diagnostic and Statistical Manual of Mental Disorders (DSM-5) criteria. Weight and height were measured in all study participants and the Body Mass Index (BMI) was calculated. In the study and control group laboratory tests assessing the liver function and the MBT were performed.

**Results:**

In all controls the anthropometric as well as laboratory liver function parameters were normal*.* In the study group 25 patients (61%) had BMI below the lower limit for age. The total percentage of ^13^CO_2_ recovery in the 120th minute of the test did not exceed the lower limit in patients and controls. A result of the ^13^CO_2_ cumulative recovery above the upper normal range was found in 18 girls with AN (44% of the study group) and 2 controls (5%). Patients with AN were characterized by significantly higher ^13^CO_2_ cumulative dose recovery after ingestion of the substrate in comparison to the control group in all time points of the test.

**Conclusions:**

The obtained results confirm a significant stimulation of the liver metabolism of ^13^C labeled methacetin in female patients with AN. The increased cumulative dose recovery of the substrate in girls with AN impacts the credibility of this measurement and implies a risk of false negative results.

## Background

Anorexia nervosa (AN) concerns approximately 0.3–4% of women, including also girls below 18 years old (around 0.8% to 1.8% of the pediatric female population) [1]. Hypetransaminasemia in the course of AN may occur as well in the period of decreased energy intake as also during realimentation [2]. Several studies in AN patients found an inverse correlation between the liver enzymes’ activity and BMI value, what suggests an important role of the nutritional status, but the mechanism of this phenomenon has not been clearly explained [3, 4]. Among possible causes cellular apoptosis and hepatocyte autophagocytosis induced by chronic malnutrition is considered. It seems probable that in the first phase of the disease, related to gradual weight loss, a moderate elevation of the activity of liver enzymes represents an attempt of the body to adapt to the decreased energy supply and protects the cells from premature death [2]. In most cases, hepatopathy in individuals with AN is mild and transient, and acute liver failure in AN is quite rare. Mild hypertransaminasemia (< 200 IU/L) concerns 30% to even 75% of AN patients [2, 5]. Usually the activity of aminotransferases exceeds the upper normal limit 2 to 4 fold, but significantly higher values can be also present [6]. As starvation continues and BMI falls below a critical value (in adults ≤13 kg/m^2^) an excessive activation of the mentioned above processes leads to destruction of hepatocytes [2]. There are few case reports of acute liver insufficiency episodes in these patients, but the total prevalence of this phenomenon is unknown [7, 8].

Another critical moment is the period of nutritional therapy with the risk of the so called refeeding syndrome (RS) as a response to the increased calories supply. Its complications cover disorders of the cardiovascular and nervous system as well as water-electrolyte imbalance and death, that may concern even 6% of adolescents hospitalized due to AN. [2]

Because of the above mentioned causes various guidelines and recommendations, including those of the American Psychiatric Association, advise constant supervision of laboratory parameters in patients with eating disorders, including even daily liver enzyme testing [9–12]. Ridout and coauthors revealed however, that such a proceeding does not translate into therapeutic decisions and generates at the same time costs and burden disproportionate to the obtained benefits [13].

Presently breath tests are increasingly applied for the assessment of the function of various organs. Substances such as ^13^C methacetin are metabolized after oral administration by the cytochrome P450. During their decomposition ^13^CO_2_ is formed and the rate of its excretion indicates the microsomal function of the liver [14]. While biochemical parameters show only hepatocyte injury and provide indirectly information about the organ function, the breath test results allow to estimate the degree of organ damage and also determine quantitatively the functional mass of the organ. Compared to the liver biopsy, which remains the gold standard of hepatological diagnostic, breath tests help to estimate not only the organ’s functional reserve but also to assess the long-term prognosis [15]. They are repeatable, easy to perform and noninvasive, what seems to be especially important in pediatric patients. However present experience concerning this method is mainly based on adult studies and only few investigated it in children. Most of the trials concerned patients with chronic, primary liver diseases or obesity [16]. Until now, to our knowledge, only results of one study on the usefulness of the breath test in the diagnostics of girls with AN were published, but the analysis was performed in a relatively small group of patients [17]. In addition this test seems to have important limitations in case of AN patients which are related to the specifics of the disease itself as well as its complications. For the above reasons this study was designed to analyze the utility of the noninvasive breath test using ^13^C carbon isotope labeled methacetin (Methacetin Breath Test - MBT) in the assessment of the liver function in girls with eating disorders of AN-type.

## Methods

### Studied groups

For the study we enrolled female patients aged 12 to 17 years, diagnosed with the restrictive type of AN (*n* = 41) and hospitalized in the Department of Children’s Endocrinology (Clinical Hospital No 1 in Zabrze of the Medical University of Silesia in Katowice) between March 2013 and May 2016. Inclusion criteria for this group were as follows: female sex, confirmed diagnosis of restrictive type of AN, and age below 18 years. Patients with cachexia in the course of other than AN organic diseases, history of a chronic liver disease, acute infection or any other than AN disease influencing the motor function of the gastrointestinal tract were disqualified from this investigation. AN was diagnosed based on the Diagnostic and Statistical Manual of Mental Disorders (DSM-5) criteria (Table 1). The medical history was additionally expanded to include information concerning the course of the main disease: the total weight loss from the beginning of the disease (in kilograms), the total weight loss (as percent of the initial weight), speed of the weight loss (in kilograms per month), duration of the secondary amenorrhea (in months, calculated based on the date of the last menstruation) and medication.

**Table 1:** Diagnostic criteria of anorexia nervosa (according to DSM-5) [4, 27]

A. Restriction of energy intake relative to requirements leading to a significantly low body weight in the context of age, sex, developmental trajectory, and physical health.	
B. Intense fear of gaining weight or becoming fat, or persistent behaviors affecting weight loss even though underweight.	
C. Disturbance in the way in which one’s body weight or shape is experienced, undue influence of body weight or shape on self-evaluation, or denial of the seriousness of the current low body weight.	
**Atypical anorexia nervosa** (AAN) – criteria B and C are met (fear of gaining weight or becoming fat, disturbance in the way in which one’s body weight or shape is experienced) and there is a significant weight loss, but the individual’s present weight is within or above the normal range for age and sex.	

The control group (*n* = 40) consisted of age-matched female patients who were hospitalized in the same time period in other pediatric departments of the Clinical Hospital No 1 in Zabrze of the Medical University of Silesia in Katowice. These girls had a normal nutritional status and - based on the anamnesis, physical examination and additional diagnostic test - eating disorders, menstruation disorders, acute infections, hepatopathy and serious organic diseases of the gastrointestinal tract were excluded.

This research project was accepted by the ethical board of the Medical University of Silesia in Katowice, Poland (KNW/0022/KBI/74/16). A written informed consent was obtained from caregivers of all the children and in case of participants older than 16 years also from the child.

### Anthropometric measurements

In all studied females anthropometric parameters (weight and height) were determined. The measurements were carried out fasting the day before the MBT. Weight was given in kilograms (with 0.1 kg precision) and height was measured with 0.1 cm precision by means of a standardized stadiometer. Based on these data for each patient the Body Mass Index (BMI) was calculated (using the equation: weight/height^2^ (kg/m^2^)). All results (weight, height, BMI) were plotted for age and sex using the percentile charts elaborated as a result of the OLA and OLAF trials (data of polish children aged 3–18 years). BMI lower or equal to the 5 percentile for age and sex was considered to be weight deficiency.

### Laboratory and other diagnostic measures

All of the study participants (patients and controls) had following laboratory tests carried out: activity of aminotransferases, including gamma glutamyltransferase, international normalized ratio (INR), serum total concentration of bilirubin and hemoglobin. Additionally girls from the study group had a cardiologic consultation with an echocardiography and the assessment of the left ventricle end-diastolic dimension (LVEDd) as well as the ejection fraction of the left ventricle of the heart (LV EF%).

### Methacetin breath test (MBT)

All patients as well as controls performed the MBT. The test was carried out using the IRIS device according to the methodology recommended by the manufacturer – the Wagner Analysen Technik Vertriebs GmbH company (Bremen, Germany) [18]. The examination was conducted in the morning in patients who remained fasting. The girls ingested 75 mg of ^13^C-methacetin (Eurisotop, France) dissolved in 200 ml of a neutral fluid at room temperature. Subsequently, according to a standardized procedure, samples of exhaled air were collected. After explaining the test’s methodology the patient was asked to take a deep breath, withhold it for 5 s and exhale the air slowly through a mouthpiece with a check valve into an aluminum covered bag filling it up completely. Following the collection of the breath sample the bag was closed tightly with a plug. Sample bags and mouthpieces used in the study were part of the equipment of the IRIS device. Air samples were collected according to the following scheme: “null” sample before the ingestion of ^13^C methacetin and subsequently 9 samples 10–20–30-40-50-60-80-100-120 min after the substrate was taken in. During the test the patients were asked to remain fasting and undertake no physical activity until the end of the procedure. All samples were analyzed by means of the IRIS device, which allowed to assess the content of ^13^CO_2_ relative to ^12^CO_2_ in the exhaled air. The increase of ^13^CO_2_ concentration compared to the baseline values was described as DOB (*delta over baseline*) and was calculated according to the formula:

$$ \mathrm{DOB}=\updelta {\mbox{\fontencoding{U}\fontfamily{wasy}\selectfont\char104}} \mathrm{PDBx}-\updelta {\mbox{\fontencoding{U}\fontfamily{wasy}\selectfont\char104}} {\mathrm{PDB}}_0 $$

where:

x – sample collected after *x* minutes.

0 – Basal “null” sample collected fasting

δ‰PDB (*Pee Dee Belemnite*) - unit determining the ^13^CO_2_ content within the total pool of the exhaled carbon dioxide relative to the international standard of the ^13^C:^12^C isotope proportion - the calcium carbonate of the fossil Belemnitella of the cretaceous Pee Dee formation is South Carolina (USA).

Based on the DOB results the software of the IRIS device calculated two basic parameters describing the kinetics of ^13^C methacetin metabolism:

1. ^***13***^***C methacetin cumulative dose (%CD) recovered with the exhaled air,*** defined as the percentage of the exhaled ^13^CO2 relative to the amount of ^13^C methacetin that was administered.
2. ***time to peak (TTP)*** – as time from the ingestion of ^13^C methacetin to the peak elimination of ^13^CO2 in the exhaled air.

Because normal ranges of MBT results for different age groups are lacking, the curves describing the kinetics of ^13^C methacetin metabolism were referred to the normative MTB ranges for healthy adults given by the device’s manufacturer.

The basic parameter to assess whether the MBT result is correct was the ^13^CO_2_ cumulative dose in the 120th minute of the test (%CD120) defined as normal when the value ranged between 20.8–37.3% of the ^13^C isotope recovery from the initially administered ^13^C methacetin dose. Additionally the kinetics of ^13^C methacetin metabolism was assessed as normal when TTP was 10–20 min.

Except for the automatic analysis, the obtained results were analyzed additionally by the authors. The main criterion of this investigation was the identification of patients whose peak ^13^CO_2_ excretion in the exhaled air was obtained after ≥20 min of the test .

### Statistical analysis

The collected data were analyzed statistically using the Statistica (StatSoft Polska Sp. z o.o.) software and Excel Microsoft Office (Microsoft Poland) worksheets. The analyses were considered as significant at *p* < 0.05. Distribution normality of the variables was verified using the Shapiro-Wilk test. Results of theses analyzes showed unequivocally normal or nearly normal distribution of the variables (*p* > 0.05). Some of the statistical calculations required standardization of variables which was performed at the beginning of the results’ elaboration. The hypotheses that there is no difference between the various tested variables describing the relationships between groups were verified by means of ANOVA analysis of the variances.

## Results

### Descriptive analysis and comparison – AN patients and control group

Inclusion criteria were met by 46 patients with AN and they performed the MBT. Out of this group 3 patients did not finish the test – two because of intended, induced vomiting after ingestion of the substrate and one due to an episode of hysteria during the test which did not allow to continue the procedure. Two other girls were excluded as a consequence of later diagnosed coeliac disease. The remaining patients (*n* = 41) comprised the study group. In the control group (*n* = 40) the MBT was carried out according to the protocol in all enrolled girls. The characteristics of both groups is presented in Table 2.

**Table 2:** Comparison of anthropometric parameters of the AN and control group

Parameter	Females with AN *n* = 41 (mean ± SD)	Control group *n* = 40 (mean ± SD)	*P*
Age (years)	14.7 ± 1.48	15.1 ± 1.62	0.258
Weight (kg)	41.23 ± 5.61	54.58 ± 6.12	< 0.001
Weight percentile	12 ± 15	53 ± 21	< 0.001
Height (cm)	162.2 ± 6.1	165.0 ± 5.3	0.030
Height percentile	48 ± 28	59 ± 26	0.084
BMI (kg/m^2^)	15.70 ± 1.84	20.08 ± 1.49	< 0.001
BMI percentile	8 ± 12	50 ± 19	< 0.001

Age of girls from the study as well as control group was comparable (*p* = 0.26). All females from the control group had normal values of anthropometric parameters (weight, height, BMI) as well as of normal results of laboratory parameters assessing the liver function – according to the initial condition for enrollment. Within the study group 25 patients (61%) were found to have BMI below the lower limit for age (mean BMI in this subgroup was 14.71 ± 1.54 kg/m^2^; mean BMI percentile - 1 ± 2). Although BMI of the other 16 patients (39%) was within the wide normal range (mean BMI in this subgroup: 17.26 ± 0.98 kg/m^2^; mean BMI percentile: 19 ± 13) they met the remaining criteria for AN diagnosis - AAN. Significant differences between the study and control group considered weight and BMI value and percentile (for all *p* < 0.001) as a result of the inclusion criteria. Among patients with AN three (7%) had abnormal AST and ALT activity and another two (5%) had isolated elevated ALT activity. In all cases the values did not exceed twice the upper normal limit. GGTP, INR and bilirubin results were within normal ranges in females from the study group. Additionally in all girls with AN, based on the echocardiography, cardiac insufficiency was ruled out. Table 3 presents the comparison of laboratory parameters characterizing both groups.

**Table 3:** Comparison of laboratory parameters characterizing the study and control group (reference values of each parameter are in the brackets)

Parameter and normal range	Patients with AN *n* = 41 (mean ± SD)	Control group *n* = 40 (mean ± SD)	*P*
Bilirubin (3.4–22 umol/l)	10,33 ± 5151	16.48 ± 13.19	0.008
AST (0–40 U/l)	24.0 ± 14.11	17.6 ± 3.06	0.007
ALT (0–41 U/l)	25.0 ± 22.13	11.9 ± 3.90	< 0.001
INR (0.9–1.3)	1.10 ± 0.083	1.02 ± 0.056	< 0.001
GGTP (10–78 U/l)	18.6 ± 14.35	12.7 ± 5.65	0.018
Hb (12–15 g/dl)	13.64 ± 1.094	13.39 ± 0.896	0.241

### MBT results

**Fig. 1:**
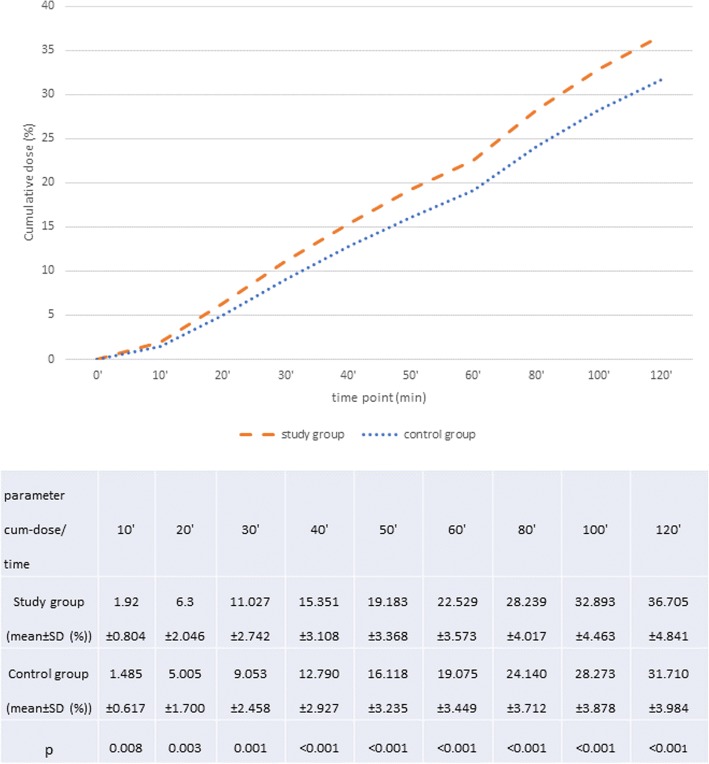
Comparison of ^13^C methacetin metabolism, presented as mean of the total percent of the ^13^CO2 dose recovery (% *cumulative dose*, CD), in the study and control group; ANOVA variance analysis (*p* < 0.05)

**Fig. 2:**
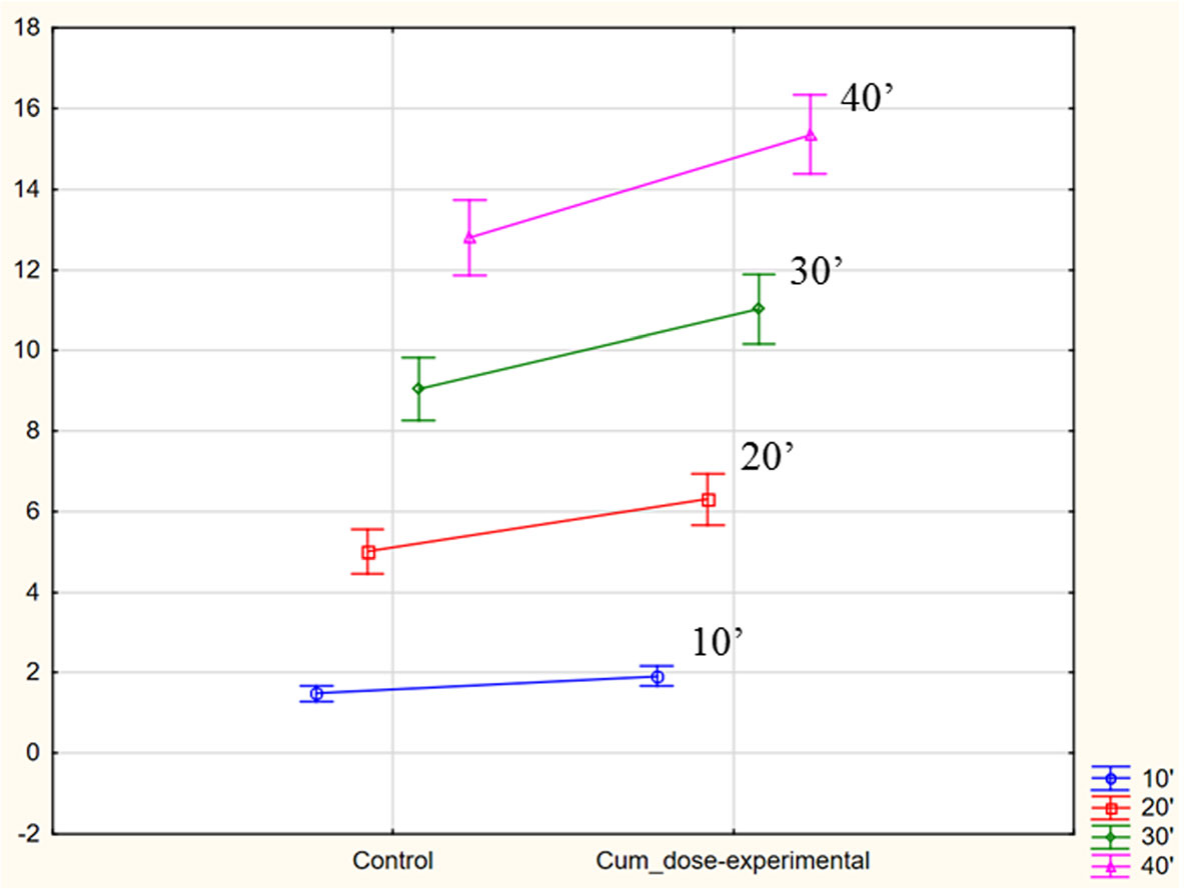
Post-hoc RIR Tukey’s test for the analyzed variable (Cum dose) 10′-40′ in the study and control group, *p* < 0.05

**Fig. 3:**
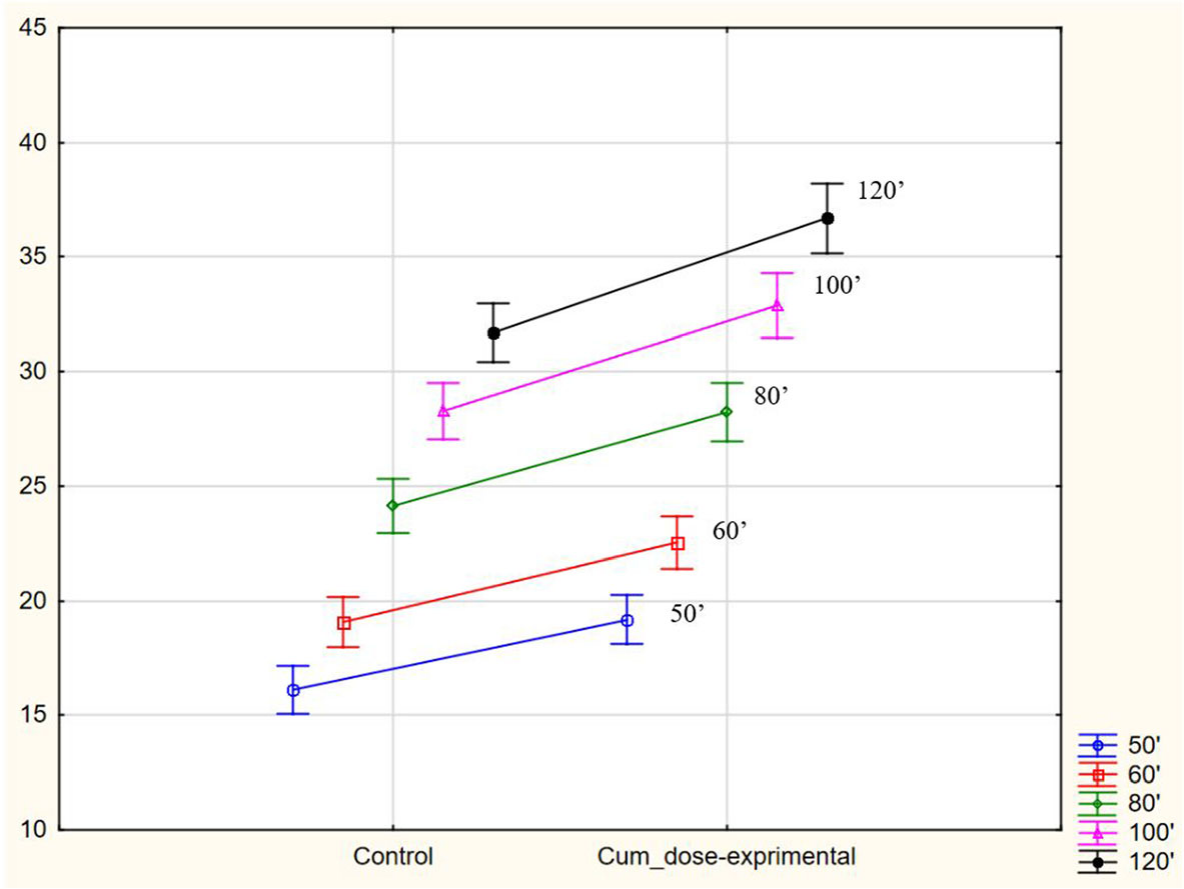
Post-hoc RIR Tukey’s test for the analyzed variable (Cum dose) 50′-120′ in the study and control group, *p* < 0.05

None of the studied individuals (in either of the groups) was found to have improperly decreased total percentage of ^13^CO2 elimination in the 120th minute of the test (%CD120 < 20.8%). Results exceeding the upper limit of the normal cumulative ^13^CO_2_ recovery (%CD120 > 37.3%) were present in 18 females with AN (43.9% of the study group) and two from the control group (5%). When compared to controls AN patients were characterized by significantly higher cumulative ^13^CO_2_ dose recovery after ingestion of the substrate. The difference concerned all time points of the test and was rising with the test’s duration. Figures 1, 2 and 3 show %CD mean and SD values in subsequent measured time points and their graphic interpretation.

**Fig. 4:**
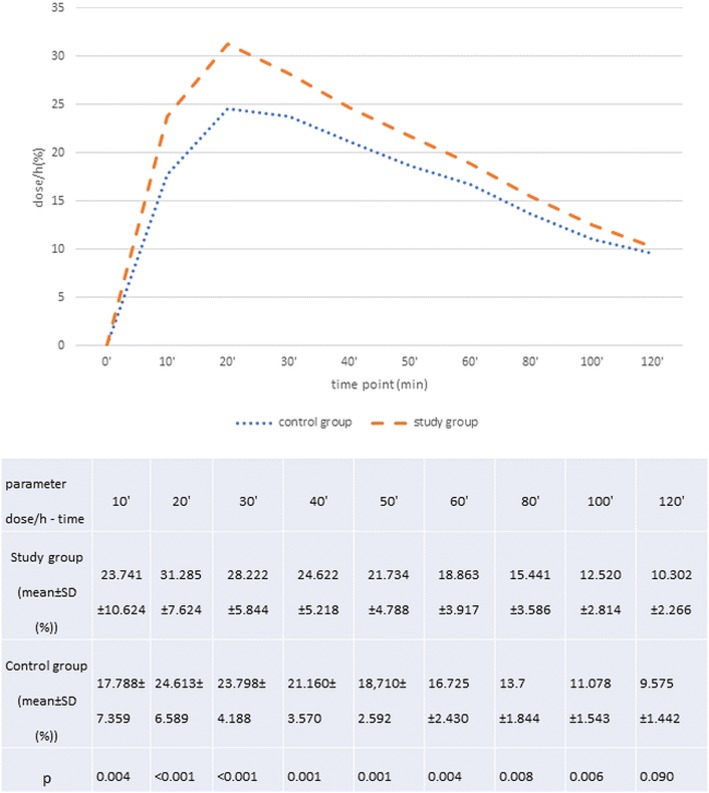
Analysis of the dose/h mean values in both groups as well as its graphic interpretation

In both, the study and in the control group, the mean ^13^CO_2_ elimination in the exhaled air (dose/h) was highest in the 20th minute of the test and it equaled 31.29 ± 7.62% in the study and 24.61 ± 6.59% in the control group respectively. Differences in mean dose/h values in individual time points between both groups and their statistical significance are presented on Fig. 4. Moreover a shift of the TTP value was observed in the two investigated groups. The majority of the patients (87.8%) and controls (95%) achieved maximal ^13^CO_2_ elimination value in the exhaled air in the measurements after ≥20 min, usually between the 20th and 40th minute of the test.

## Discussion

Hypertransaminasemia is one of the most common liver phenomenon observed in AN patients [6]. Within our study group hypertransaminasemia was revealed in 12.2% females with AN, wherein accordingly to data published by other authors, slightly higher ALT than AST activity was observed (24.99 IU/L ± 22.13 and 23.99 ± 14.11 IU/L respectively). None of the studied patients presented clinical or biochemical signs of liver insufficiency and the highest values of the liver enzymes activity did not exceed more than 3 fold the upper normal limit.

Application of the MBT in the assessment of the liver function is a promising diagnostic option, which is especially interesting in pediatrics, particularly in groups requiring constant or repeated monitoring of this organ’s function. MBT is indisputably one of the best described metabolic breath tests. Although the efficiency of MBT in the diagnostics of liver function was confirmed in different diseases, also in children, many factors may negatively influence the investigative value of this test. In the case of AN already the exactness of the execution of the test’s methodology may rise doubts. The test’s protocol assumes efficient consumption of 75 mg of ^13^C methacetin (odorless powder) dissolved in 200 ml of neutral, room-temperature fluid. Taking into consideration the psychopathology of the illness, ingestion of such amount of fluid may be difficult or even traumatizing for the patient. In two of our patients vomiting occurred soon after the ingestion of the substrate and test had to be discontinued. One girl could not consume the substrate because of a hysteria episode. Another limitation may be the duration of the test. During the 120 min test time the patients must remain fasting what may be in opposition to the refeeding plan whereas, in this group the nutritional refeeding should be priority.

Apart from psychological limitations also gastroenterological aspects of AN may play an important role. In AN patients such disorders as regurgitations, dysphagia, odynophagia, nausea or gastro-esophageal reflux can be observed [19]. These symptoms may lead to difficulties in the intake of ^13^C methacetin or ingestion of an inadequate amount of the substrate. Moreover AN is related to disorders of the motility of the gastrointestinal tract, including delayed gastric emptying and prolonged gut passage [19]. In our studied group of AN patients TTP was longer compared to normal ranges given by the manufacturer, what may be caused by the delayed passage and – in consequence – postponed absorption of the substrate. In the study group the maximal mean value of dose/h was in the 20th minute of the test. The time from ^13^C methacetin administration to the peak excretion of ^13^CO_2_ in the exhaled air was usually between the 20th and 40th minute of the test (normal range: 10th–20th minute of the test). A similar situation was revealed also in the control group. It needs to be however underlined that the presumed normal ranges refer to measurements in adults, because there are no separate normal/reference values for the pediatric population. This fact might influence the present observations. Piwczyńska and coauthors, who investigated MBT utility in a group of children with autoimmune hepatitis, described significantly longer TTP in patients with severe liver fibrosis. The authors’ explanation of these results was the loss of metabolically active liver tissue during fibrosis [20]. Similar observations were described by Shteyer and coauthors who found significantly longer TTP in infants with biliary atresia. According to the authors it reflected the decreased activity of the hepatic P450 cytochrome in this group [21]. In our patients with AN, despite prolonged TTP, the mean maximal percentage for dose/h which describes the recovery of the substrate, was high - significantly higher than in the control group (31.29 ± 7.62% vs 24.61 ± 6.59%; *p* = 0.000). This finding may suggest rather stimulation of the hepatic methacetin metabolism and a different than liver insufficiency cause of delayed TTP.

In an earlier study that assessed liver function in girls with AN by means of MBT significant acceleration of the liver ^13^C methacetin metabolism was described when compared to the control group and the differences in %CD values between children with AN as controls were significant for all measurements after the 30th minute of the test. The authors’ interpretation of the obtained data was that girls with AN have a specific model of ^13^C methacetin metabolism which is related to, among others, increased recovery of the cumulative dose of the substrate. The mentioned study was however performed in a small group of children (*n* = 25, only 12 with AN) what importantly limited the reasoning power [17]. Our investigation of over 80 females confirms the significantly higher ^13^CO_2_ cumulative dose values in the exhaled air in the study group in comparison to controls. Statistically significant differences concerned in this case all measurement time points. The dissimilarity of the methacetin metabolism in the group of AN patients could on one side evidence an actual increase of the liver’s cytochromal activity, but on the other side it may reflect the influence of exogenous factors on the measurement results. Existing studies on the impact of malnutrition on the activity of the liver P450 cytochrome were conducted in various age groups using diverse methodology and, in consequence, present ambiguous results. Hamon-Wilcot and coauthors investigated the activity of the CYP1A2 subunit of the P450 cytochrome in individuals above the age of 65 years with weight loss and hypoalbuminemia. The activity of the cytochrome was assessed using the paraxanthine/caffeine metabolic index. Its values in the study and control group were similar. There were also no correlations between BMI and the CYP 1A2 subunit activity [22]. On the other hand Oshikoya and coauthors by means of a ^13^C caffein breath test (CBT) proved significantly decreased recovery of the cumulative dose of the substrate (%cum dose) in malnourished children in the course of marazmus and kwashikor in comparison to the test’s results that were obtained after realimentation [23]. The outcomes of the last of the mentioned studies seem to stay in opposition to our observations. Nonetheless it needs to be pointed out, that in contrast to methacetin, caffeine is a substance characterized by a low hepatic extraction index and the CBT is considered to be relatively resistant to exogenous factors [24]. Therefore it seems to be essential to separate the impact of weight and nutritional status from other factors related to AN that might influence the MBT result. Studies analyzing the relations between the liver’s functional capacity and the left ventricle ejection fraction (LV EF) as well as the dimensions of the left heart showed a correlation between %CD of ^13^C methacetin in the 120th minute of the test and the stage of heart insufficiency (according to NYHA). The value of the ^13^CO_2_ CD was also significantly lower in all time points in patients with lower LV EF. %CD ^13^CO_2_ weekly correlated with LV EF as well as it had a week invert association with the LVEDd [25]. Our studied AN patients did not reveal signs of heart insufficiency. Their mean LV EF was 69.14 ± 5.092% and mean LVEDd – 4.202 ± 0.355 cm. This however does not rule out the possible impact of a single LV EF value on the obtained %CD^13^CO_2_. The observed acceleration of the hepatic methacetin metabolism in the study group may also be a consequence of the patients’ medication. At the same time the increased liver metabolism of the CYP1A subunit could in a difficult to predict way modify the pharmacokinetics of the drugs used in AN treatment.

Undoubtedly an important role in the interpretation of the obtained results play the selection criteria for the study group. Tsukamoto and coauthors investigated 25 adult patients with eating disorders before realimentation was started. Elevated ALT activity was found in 13 of them and these were patients with confirmed AN. Individuals with incorrect ALT values had lower BMI and shorter disease duration. The BMI value was considered to be a significant and independent determinant in the process of liver injury in patients with AN [26]. Similar observations were conducted by Hanachi and coauthors, who were looking for risk factors of hypertransaminasemia in a cohort of AN patients obtaining nutritional therapy [3]. In our study we used the BMI percentile values in order to compare children of different age. The mean BMI percentile in the study group was 7.959 ± 11.696, with the values varying from the 50th below the 1st percentile for age. This wide range of this parameter results from the fact, that the study group included also girls with so called AAN (who fulfill the B and C criteria of the DSM-5 classification accompanied by a significant weight loss, but whose present weight was within normal ranges for age and sex). Patients with AAN have typically higher weight (at the beginning of the disease even obesity) and more intensive nutritional restrictions compared to typical AN, but the risk of organ complications is in both cases is similar [4]. On one hand it can be concluded, that higher BMI in girls with AAN, being an indirect evidence of better nutritional status, is related more to adaptive changes of the liver metabolism and to the domination of cytoprotective over cytolytic processes leading to the preservation of the normal liver function. However on the other hand females with AAN usually undertake more intensified actions aimed at fast reduction of the weight, what combined with the typical for adolescents high basic metabolism index, may lead to earlier development of metabolic complications [3]. This group may therefore have a difficult to foresee liver metabolism status which influence the general outcomes of our study. The multiplicity of the presented above factors that may potentially interfere with the MBT results casts in doubt the usefulness of this method in patients with eating disorders of AN type.

The practical implication of our finding is that described by us peculiar feature of MBT in AN patients may decrease the test’s sensitivity in this group. The percentage of the exhaled ^13^CO_2_ cumulative dose in the 120 min of the test is one of the basic parameters in result interpretation. Higher %CD 120, due to increased liver cytochromal activity, result in normal test outcomes and create a risk of false negative results. A potentially higher percentage of false negative results significantly decreases the diagnostic value of this method. Nevertheless, this study has also some limitations that require mentioning. The study group included only mild hypertransaminasemia patients and therefore it is difficult to predict if the findings would be similar in AN individuals with severe hypertransaminasemia and/or liver failure. The background of our outcomes is still unclear. Two factors that may influence the MBT results in AN patients need to be considered. Firstly, it seems to be critical to estimate if the test results depend on weight and nutrition status of the patient. Secondly it is crucial to exclude other than undernutrition factors that can impact the test’s results. A prospective study would be helpful to analyze whether the methacetin metabolism changes after realimentation.

The strengths of the study include its case-control design that allowed to compare MBT parameters in AN patients with sex and age-matched children. We also excluded heart failure in patients from the study group - one of the most important factors that influence the MBT results. Furthermore to the best of our knowledge this is the first study that estimates the utility of ^13^C MBT in a representative group of patients with eating disorders of AN type.

## Conclusions

The obtained results confirm that the liver ^13^C methacetin metabolism in female patients with AN is significantly increased in comparison to healthy controls. These observations, apart from clinical implications related to the possible increased activity of the enzymatic system of the liver P450 cytochrome, generate also a question concerning the usefulness of the MBT in the assessment of the liver function in this specific group of patients. This method still raises many interpretational doubts. Increased cumulative dose recovery of the substrate in girls with AN influences the reliability of the result. The described phenomenon creates also a risk of obtaining a higher percentage of false negative results, what significantly decreases the diagnostic value of this method. There is a need for further clinical studies that would assess the influence of exogenous factors related to AN on the test’s result.

## Abbreviations

AAN: Atypical anorexia nervosa
AN: Anorexia nervosa
BMI: Body mass index
CBT: Caffein breath test
CD: Cumulative dose
DSM: Diagnostic and Statistical Manual of Mental Disorders
GGTP: Gamma glutamyltransferase
Hb: Hemoglobin
INR: International normalized ratio
LV EF: Left ventricle ejection fraction
LVEDd: Left ventricle end-diastolic dimension
MBT: Methacetin breath test
RS: Refeeding syndrome
TTP: Time to peak
